# Proteomic analysis of spinal cord tissue in a rat model of cancer-induced bone pain

**DOI:** 10.3389/fnmol.2022.1009615

**Published:** 2022-12-05

**Authors:** Heyu Yang, Ji Wu, Shuqing Zhen, Yindi Hu, Dai Li, Min Xie, Haili Zhu

**Affiliations:** ^1^Xianning Medical College, Hubei University of Science and Technology, Xianning, China; ^2^Affiliated Hospital of Youjiang Medical University for Nationalities, Baise, China; ^3^Matang Hospital of Traditional Chinese Medicine, Xianning, China

**Keywords:** cancer-induced bone pain, spinal cord, LC-MS/MS, differentially expressed protein, synapse

## Abstract

**Background:**

Cancer-induced bone pain (CIBP) is a moderate to severe pain and seriously affects patients’ quality of life. Spinal cord plays critical roles in pain generation and maintenance. Identifying differentially expressed proteins (DEPs) in spinal cord is essential to elucidate the mechanisms of cancer pain.

**Methods:**

CIBP rat model was established by the intratibial inoculation of MRMT-1 cells. Positron emission tomography (PET) scan and transmission electron microscopy (TEM) were used to measure the stats of spinal cord in rats. Label free Liquid Chromatography with tandem mass spectrometry (LC-MS-MS) were used to analyze the whole proteins from the lumbar spinal cord. Differentially expressed proteins (DEPs) were performed using Gene Ontology (GO) and Kyoto encyclopedia of genes and genomes (KEGG) enrichment analysis, and verified using Western blot and immunofluorescence assay.

**Results:**

In the current study, CIBP rats exhibited bone damage, spontaneous pain, mechanical hyperalgesia, and impaired motor ability. In spinal cord, an hypermetabolism and functional abnormality were revealed on CIBP rats. An increase of synaptic vesicles density in active zone and a disruption of mitochondrial structure in spinal cord of CIBP rats were observed. Meanwhile, 422 DEPs, consisting of 167 up-regulated and 255 down-regulated proteins, were identified among total 1539 proteins. GO enrichment analysis indicated that the DEPs were mainly involved in catabolic process, synaptic function, and enzymic activity. KEGG pathway enrichment analysis indicated a series of pathways, including nervous system disease, hormonal signaling pathways and amino acid metabolism, were involved. Expression change of synaptic and mitochondrial related protein, such as complexin 1 (CPLX1), synaptosomal-associated protein 25 (SNAP25), synaptotagmin 1 (SYT1), aldehyde dehydrogenase isoform 1B1 (ALDH1B1), Glycine amidinotransferase (GATM) and NADH:ubiquinone oxidoreductase subunit A11 (NDUFA11), were further validated using immunofluorescence and Western blot analysis.

**Conclusion:**

This study provides valuable information for understanding the mechanisms of CIBP, and supplies potential therapeutic targets for cancer pain.

## Introduction

Cancer is one of the leading causes of death worldwide and seriously impacts the health-related quality of life in patients. According to the statistics in 2022, there will be approximately 4,820,000 new cancer cases and 3,210,000 cancer deaths in China (Siegel et al., 2022). Metastases, not the primary tumor, are responsible for 90% of cancer deaths, and bone is a frequent site of metastases (Zhang et al., 2022). About 75–90% of patients with metastases develop moderate or severe pain (Glithero, 2020), and more than 80% of cancer pain is attributed to metastatic cancer-induced bone pain (CIBP) (Wang et al., 2020). CIBP has elements of acute, inflammatory, and neuropathic pain, in a crippling, chronic, morbid state, and significantly decreases patients’ functional capacity (Zaja̧czkowska et al., 2019). According to the World Health Organization (WHO) analgesic ladder, CIBP management uses non-steroidal anti-inflammatory drugs (NSAIDs), paracetamol, and opioids (alone or in combination). The adverse effects of NSAID treatment include gastrointestinal irritation/bleeding, kidney dysfunction, cardiovascular events, or bleeding disorders. For opioid treatment, the side effects include physical dependence, tolerance, and respiratory depression (Ellingson and Vanderah, 2020). Until now, more than half of patients with metastatic cancer receive inadequate treatment and suffer from pain (Wang et al., 2021). Moreover, the pathological mechanisms of CIBP are complex involving tumor cells, bone cells, inflammatory microenvironment, and neuronal tissue, and still unclear (Kapoor et al., 2021). Therefore, elucidation of the pathogenesis of CIBP helps explore novel therapies for pathological pain.

Growth of metastatic tumor cells in bone marrow stimulates osteolysis, aggravates bone destruction, injuries the peripheral nerves, and triggers the transduction of peripheral nociceptive signals (Julius and Basbaum, 2001). These nociceptive signals reach to spinal dorsal horn, subsequently transmit to the brain through spinal ascending pathways, and consequently generate neuropathic pain (Ban et al., 2021; Tilley et al., 2021). As a critical center for nociceptive processing, the spinal cord undergoes significant functional abnormalities and sensitization (Garcia et al., 2022), characterized as two common clinical features, such as pathological amplify of noxious and innocuous stimuli, and persistent hyperalgesia and allodynia (Kuner, 2010). In patients with fibromyalgia syndrome which is a debilitating chronic pain condition, spinal dorsal horn activity is enhanced using functional magnetic resonance imaging (fMRI) (Kornelsen and Mackey, 2007; Bosma et al., 2016). In cancer-pain mice model, spinal synaptic transmission mediated through A-delta and C-fibers is enhanced (Yanagisawa et al., 2010). Moreover, electrical epidural spinal cord stimulation is considered a powerful neuromodulation tool for treating chronic pain (Laakso et al., 2021). It is reported that spinal cord stimulation reduces patients’ painful symptoms and improves their quality of life (Paolini et al., 2022). Accordingly, the determination of key proteins for modulating nociceptive signals in the spinal cord could be an essential strategy for CIBP therapy.

Proteomics techniques are used for the large-scale study of changes in protein expression. To figure out the factors that participate in spinal sensitization and cancer pain, label-free LC-MS/MS was applied to analyze the changes in spinal proteins in CIBP rats. Consequently, the differentially expressed proteins (DEPs) were identified. Gene Ontology (GO) and Kyoto Encyclopedia of Genes and Genomes (KEGG) enrichment analysis were performed. Western blot and immunofluorescence assay were used to verify protein expressions. Our research provides the basis for the mechanism of cancer pain and targets analgesic treatment.

## Materials and methods

### Experimental animals and groups

Male Sprague-Dawley (SD) rats (180∼200 g) were purchased from Hubei Province Experimental Animal Center (Wuhan, CHN) and housed under controlled conditions (22 ± 1°C, 12 h alternating light–dark cycle) with free access to water and food. All housing, breeding, and procedures were performed according to the local and international guidelines for the Care and Use of Experimental Animals and approved by the Ethics Committee of Hubei University of Science and Technology (2019-03-021). Animals were randomly divided into two groups, such as the control group and the CIBP group (nine per group).

### Establishment of cancer-induced bone pain rat model

Rats were anesthetized by pentobarbital sodium (50 mg/kg, intraperitoneal). Left legs were shaved and disinfected with 75% (v/v) ethanol. The suspension of 5.0 × 10^5^ MRMT-1 cells was slowly injected into the intramedullary cavity of the tibia (Yao et al., 2019). The sham group applied the same operation with the injection of Hank’s solution. The syringe was detained for at least 1 min to prevent the leakage of tumor cells. After that, the injection site was sealed with bone wax as soon as the syringe was removed. The wound was then stitched with silk thread.

### Radiology

To confirm tibial destruction, radiographic examination of the ipsilateral tibia was performed on the 14th day following tumor cell inoculation. The hind limbs were isolated from the body, shed most cutaneous tissue and muscle, placed on X-ray film, and exposed to an X-ray source. Radiographic images were captured.

### Bone histology

Tibia was harvested, fixed in 4% paraformaldehyde (PFA), and decalcified in a decalcification solution. Then, the decalcified bones were rinsed, dehydrated, embedded in paraffin, and sectioned into 4 μm sections (Thermo Fisher, USA). The sections were deparaffinized, rehydrated, and conducted to hematoxylin and eosin (H&E) staining (Beyotime, CHN) according to standard procedures. The stained sections were observed using a microscope (Olympus IX73, JPN) (Li et al., 2019).

### Spontaneous pain

The number of spontaneous flinches was recorded to assess spontaneous pain (Liu et al., 2021). Rats were placed in a 30 cm × 30 cm × 30 cm transparent acrylic box to adapt for 30 min. The number of spontaneous flinches (foot retraction or licking) of rats was recorded every 5 min for three times. The number of spontaneous flinches was examined during pre-surgery (day 0) and on days 0, 4, 7, 10, and 14 after surgery.

### Mechanical allodynia

Mechanical allodynia was evaluated by the measurement of ipsilateral hind paw withdrawal threshold (PWT) in response to von Frey filament stimuli. Rats were placed on a 5 × 5 mm wire mesh grid floor and allowed to acclimate for 30 min before testing. The von Frey filaments ranging from 0.4 to 26 g (Stoelting, USA) were applied on the mid-plantar of the left hind paw for 3–5 s per filament with a 2-min interval between applications. The calibrated monofilaments were applied perpendicularly to the plantar surfaces until the filaments were bent. Brisk withdrawal or paw flinching upon stimulus was considered positive response. When a positive response occurred, the next lower force of the von Frey filament was used. Conversely, the next higher force of filament was used. Finally, the pattern of positive and negative withdrawal response was converted to 50% PWT (Dixon, 1980).

### Rotarod test

The motor coordination and balance of rats were tested by the rotarod test. Before the test, rats were placed on a rotarod device (ZS-RDM-XS, China) and allowed to explore the rotarod three times (10 min/time) with a constant speed of 4 r/min, and rest for 30 min at an interval. The rod was rotated from a speed of 10 to 20 rpm for 10 min (Li et al., 2020). The latency to fall, which is the cumulative time (s) that each animal remained on the rotating rod, was recorded as an indicator of its motor coordination (Shi et al., 2021).

### ^18^F-FDG positron emission tomography imaging

Before PET imaging, rats were fasted for 12 h, anesthetized with 2% isoflurane, and injected with a dose of approximately 500 ± 25 μCi of 18-fluorodeoxyglucose (FDG) *via* tail vein. After 50 min of FDG uptake, rats were placed on a scanning bed for a PET scan. PET images were obtained with the whole-body mode (scan beds: 2; scan times for each bed: 7 min) for 14 min by the TransPET Discoverist 180 system (Raycan Technology Co., Ltd., Suzhou, China). The PET images were reconstructed using the three-dimensional (3D) OSEM method with a voxel size of 0.5 × 0.5 × 0.5 mm^3^. The mean standardized uptake value (SUV) was calculated using the following formula: mean pixel value with the decay-corrected region-of-interest activity (μCi/kg)/[injected dose (μCi)/weight (kg)] (von Leden et al., 2016).

### Spinal cord histology

At 14 days after cell inoculation, rats were anesthetized with pentobarbital sodium (50 mg/kg, intraperitoneal), immediately perfused with ice-cold saline, and fixed with 4% paraformaldehyde. Then, the retrieved spinal cord tissue was embedded in paraffin and cut into 4 μm sections. After the deparaffinization and rehydration, the spinal cord sections were stained with an H&E Staining Kit or Nissl Stain Solution (Solarbio, CHN), according to the standard procedures. All stained sections were observed under a microscope. The inflammatory score and numbers of Nissl body were analyzed by ImageJ software (Dong et al., 2022).

### Western blotting

Rats were anesthetized deeply and euthanized by decapitation. Spinal cord tissues were collected and lysed with ice-cold RIPA buffer containing protease and phosphatase inhibitor (Sigma, USA) for 30 min, and centrifuged at 12,000 r/min for 15 min. After centrifugation, the supernatant was collected, mixed with loading buffer, and heat-denatured at 95°C for 10 min. The protein concentration was determined by a bicinchoninic acid (BCA) analysis kit (Beyotime, China). Equal amounts of protein samples were separated in SDS-PAGE and transferred to PVDF membranes. The membranes were blocked with blocking buffer (Beyotime, China) at room temperature for 60 min, incubated with the appropriate primary antibodies at 4°C overnight and HRP-conjugated secondary antibodies at room temperature for 60 min, and finally visualized using enhanced ECL chromogenic solution under iBright 1500 imaging system (Invitrogen, USA). The following antibodies from ABclonal Technology were used: CPLX1 rabbit pAb (1:1000, A11588), SNAP25 rabbit pAb (1:1000, A2234), SYT1 rabbit pAb (1:1000, A0992), ALDH1B1 rabbit pAb (1:1000, A3725), GATM rabbit pAb (1:1000, A6598), NDUFA11 rabbit pAb (1:1000, A16239), and HRP-conjugated secondary antibodies (1:5000, AS014).

### Immunofluorescence

Spinal cord tissues were fixed with 4% paraformaldehyde, embedded in paraffin, and sectioned to 4 μm. After the deparaffinization and rehydration, the spinal cord sections were immersed in citrate antigen retrieval solution (Beyotime, China) for antigen repair, incubated with 3% hydrogen peroxide for 10 min blocked with Immunol Staining Blocking Buffer (Beyotime, China) for 60 min, incubated with anti-SNAP25 rabbit pAb (A2234, ABclonal), GATM rabbit pAb (1:1000, A6598), and NDUFA11 rabbit pAb (1:1000, A16239) overnight at 4°C and goat anti-rabbit IgG H&L (FITC) (ab6717, USA) at room temperature for 60 min. The images were captured with a fluorescence microscope (Olympus IX73, JPN). The relative fluorescence intensities were analyzed by ImageJ software (Yu et al., 2020).

### Transmission electron microscopy

Ipsilateral of lumbar spinal cord tissues was isolated, cut into ∼1 mm^3^ cubes, fixed in 2.5% glutaraldehyde, and post-fixed with 1% osmium tetroxide. Ultrathin sections were post-stained with uranyl acetate and lead citrate and then examined using an HC-1 transmission electron microscope (Hitachi, Japan) operating at 120 kV (Chen et al., 2017).

### Liquid chromatography-tandem mass spectrometry

Three samples of the ipsilateral lumbar spinal cord from sham or CIBP rats were mixed and whole proteins were extracted as follows: tissues were triturated with liquid nitrogen, lysed by sonication with quadruple of phenolic extraction buffer (including 10 mM dithiothreitol, 1% protease inhibitors, and 2 mM EDTA), added equal volume of Tris–phenol solution, and centrifugated at 5,500 g for 10 min at 4°C. The supernatants were treated with the quintuple of volumes 0.1 M ammonium acetate/methanol to sediment overnight. The precipitations were obtained, washed with methanol and acetone, and dissolved using 8 M urea. The samples were concentrated using the BCA kit and digested with trypsin overnight at 37°C. The digested peptides were freeze-dried under vacuum, dissolved into mobile phase A (including 0.1% formic acid and 0.2% acetonitrile), and separated into 60 fractions using Agilent 300 Extend C18 column (4.6 × 250 mm, 5 μm, model number: 770995-902) and a UPLC system with increasing mobile phase B (20 mM formic acid in acetonitrile) from 5 to 35% over 60 min. The fractionated peptides were injected into an NSI ion source for ionization and analyzed by Q Exactive HF mass spectrometry (MS, model number: 0726090). MS data were acquired across the mass range of 350–1,200 m/z in high-resolution mode (70,000) with 250 ms accumulation time per spectrum and explored to qualify in the protein database. Listed proteins were searched using the analysis software PEAKS and matched with various databases. The identified protein sequences were derived from the UniProt database (Zhao et al., 2016).

### Gene ontology and kyoto encyclopedia of genes and genomes enrichment analysis of clusters

An R package, clusterProfiler, was used to perform GO, KEGG enrichment analysis of clusters, and the ggplot2 package to visualize the results. All proteins were grouped into three major categories: biological processes (BPs), cellular components (CCs), and molecular functions (MFs). The Kyoto Encyclopedia of Genes and Genomes (KEGG) was employed to predict metabolic pathway classification (Lei T. et al., 2021).

### Protein–protein interaction analysis

The identified differential expression proteins were imported into the STRING database^1^ to construct a (Protein–Protein Interaction) PPI network, and the confidence score of the minimum required interaction score was set greater than or equal to 0.7. The PPI network was visualized using Cytoscape (Doncheva et al., 2019).

### Statistical analysis

All statistical analyses were conducted with SPSS 21.0, and the R (version 4.1.0) analysis of variance (one-way ANOVA) was used for the comparison of values between different experimental groups, followed by Bonferroni *post-hoc* tests. A value of *p* < 0.05 was considered statistically significant. Data from behavioral tests were shown as mean ± SEM. PET data, histology data, immunofluorescence data, and Western blot data were shown as mean ± SD.

## Results

### Spinal cord undergoes functional abnormalities in cancer-induced bone pain rats

**FIGURE 1:**
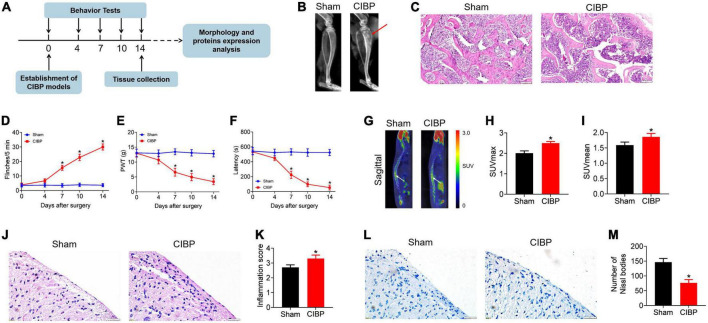
Validation of the cancer-induced bone pain (CIBP) rat model and the characterization of spinal functional changes. **(A)** Schematic diagram of the experimental procedures. On day 0, the CIBP model was established. On days 0, 4, 7, and 14 after surgery, the behavioral tests including spontaneous flinches record, PWT value test, and latency to fall assay were performed. On day 14, X-ray scan, PET scan, histology staining, Western blotting, and LC-LC/MS assay were performed after behavioral tests. **(B)** Representative radiographs of the ipsilateral tibia from sham and CIBP rats on day 14. The red arrows indicate areas of bone destruction in CIBP rats (*n* = 9 per group). **(C)** Representative images of H&E staining of tibial sections from sham and CIBP rats (*n* = 3 per group). Scale bars: 100 μm. **(D)** The numbers of spontaneous flinches of sham and CIBP rats. Data were expressed as the mean ± SEM (*n* = 9 mice/group). *p < 0.05 vs. sham group. **(E)** PWT values were assessed by von Frey filaments with the up-and-down method. Data were expressed as the mean ± SEM (*n* = 9 mice/group). *p < 0.05 vs. sham group. **(F)** The latency to fall of accelerated rotarod motor test of sham and CIBP rats. Data were expressed as the mean ± SEM (*n* = 9 mice/group). *p < 0.05 vs. sham group. **(G–I)** Representative ^18^F-FDG PET images in spinal cord (sagittal) **(G)**. SUVmax **(H)** and SUVmean **(I)** of the spinal cord from sham and CIBP rats. Data were expressed as the mean ± SD (*n* = 3 mice/group). *p < 0.05 vs. sham group. **(J–K)** Representative images **(J)** and quantitative analysis **(K)** of H&E staining of the spinal cord. Scale bar: 20 μm. Data were expressed as the mean ± SD (*n* = 3 mice/group). *p < 0.05 vs. sham group. **(L,M)** Representative images **(L)** and quantitative analysis **(M)** of Nissl bodies for spinal and quantitative analysis **(M)**. Data were expressed as the mean ± SD (*n* = 3 mice/group). *p < 0.05 vs. sham group.

According to the experimental protocol shown in Figure 1A, the CIBP rat model was established by the intratibial inoculation of MRMT-1 cells on day 0. Behavioral tests including spontaneous flinches record, PWT value test, and latency to fall assay were performed on days 0, 4, 7, and 14 after surgery. X-ray scan, PET scan, histology staining, Western blotting, and LC-LC/MS assay were performed on day 14. As a result, X-ray tibia radiographs showed significant bone destruction and uneven local bone density in CIBP rats. However, no abnormalities were observed in the tibia of sham rats (Figure 1B, n = 9). H&E staining of the tibia observed a defection of trabecular bone in CIBP rats, while the intact trabecular bone was in the sham group (Figure 1C, n = 3). On days 7, 10, and 14, the numbers of spontaneous flinches indicating spontaneous pain significantly increased to 16.00 ± 2.52, 23.16 ± 2.61, and 31.16 ± 1.97 in CIBP rats (*p* < 0.05 vs. sham, Figure 1D, n = 9). PWT values presenting mechanical pain sensitivity were dramatically decreased to 5.58 ± 1.10, 3.98 ± 1.06, and 3.04 ± 1.12 in CIBP rats (*p* < 0.05 vs. sham, Figure 1E, n = 9). The latency presenting motor coordination and balance were markedly reduced to 178.46 ± 41.21, 97.39 ± 31.32, and 46.53 ± 28.43 in CIBP rats (*p* < 0.05 vs. sham, Figure 1F, n = 9). No significant changes in flinches numbers, PWT values, and latency were detected in sham rats (*p* > 0.05, Figures 1D–F, *n* = 9). These results indicated that the inoculation of tumor cells resulted in a nociceptive effect.

Positron emission tomography scan and histology analysis were performed to measure the changes in the spinal cord in CIBP rats. ^18^F-fluoride is a positron-emitting isotope with high specificity for sites of bone turnover (Mathavan et al., 2019). PET with ^18^F-FDG is a diagnostic tool to evaluate metabolic activity by measuring FDG accumulation (Kawada et al., 2016). In this study, the ^18^F-FDG PET scan was used to visualize metabolic activity in the spinal cord and PET images revealed that the PET signal was significantly increased in CIBP rats (*p* < 0.05 vs. sham, Figure 1G, n = 3). The maximum SUV (SUVmax) and mean SUV (SUVmean) of the spinal cord in sham and CIBP rats were 2.01 ± 0.11 vs. 2.5 ± 0.1 (*p* < 0.05 vs. sham, Figure 1H) and 1.59 ± 0.1 vs. 1.86 ± 0.11, respectively (*p* < 0.05 vs. sham, Figure 1I). The result demonstrated prominently hypermetabolic lesions in the spinal cord of CIBP rats. Histology analysis of the spinal cord was detected by H&E staining and Nissl staining. Severe leukocyte infiltration was observed in the spinal dorsal horn of CIBP rats detected by H&E staining (Figure 1J). The inflammation scores in sham and CIBP groups were 2.7 ± 0.17 and 3.3 ± 0.23 (*p* < 0.05 vs. sham group, *n* = 3, Figure 1K). The number of Nissl bodies in gray matter of spinal dorsal horn from CIBP rats was significantly reduced detected by Nissl staining (Figure 1L), indicating an injured ability of protein synthesis and release. Nissl-positive cells in sham and CIBP groups were 146.33 ± 12.42 and 76.66 ± 10.96, respectively (*p* < 0.05 vs. sham group, *n* = 3, Figure 1M). Taken together, bone metastasis of cancer cell induced the functional abnormality of the spinal cord.

### Global proteomic changes in the spinal cord of cancer-induced bone pain rats

**FIGURE 2:**
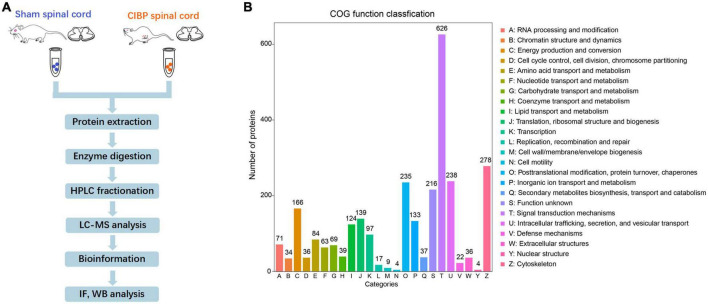
Global proteomic identification in the spinal cord of sham and cancer-induced bone pain (CIBP) rats using liquid chromatography with tandem mass spectrometry (LC-MS/MS). **(A)** Schematic diagram of the experimental procedures. **(B)** Functional classification of proteins by COG. X-axis: different classifications. Y-axis: the number of proteins in each class. COG: Cluster of Orthologous Groups of proteins.

To accurately quantify the proteomic changes in the spinal cord of CIBP rats, protein extracts were analyzed by LC-MS/MS. The experiment protocol is shown in Figure 2A. The proteomic analysis of extracts from the spinal cord identified a total of 1,539 proteins. COG protein database was used to predict the biological function of proteins and the results showed that all annotated proteins were classified into 24 clusters (Figure 2B). The three largest groups of the functional classification were signal transduction mechanisms (626 proteins), cytoskeleton (278 proteins), intracellular trafficking, secretion, and vesicular transport (238 proteins). Among these proteins, 422 proteins were identified as statistically differentially expressed proteins (DEPs) in the spinal cord between sham and CIBP rats. The 167 proteins were significantly up-regulated with fold change ≥ 2 and *p-*value ≤ 0.05, while 255 proteins were down-regulated with fold change ≤ 0.5 and *p-*value ≤ 0.05. The top 30 up-regulated and 30 down-regulated proteins are shown in Tables 1, 2, respectively.

**TABLE 1:** The detail information of the top 30 up-regulated differentially expressed proteins.

Accession	Protein	Fold change (CIBP/Sham)	Log2 fold change (CIBP/Sham)	Regulation
P63041	Cplx1	12.8	3.7	Up
P84087	Cplx2	12.8	3.7	Up
Q1AAU6	Asap1	10.2	3.3	Up
P69682	Necap1	9.7	3.3	Up
P21139	Man2c1	9.6	3.3	Up
Q6P7A2	Ube4a	9.4	3.2	Up
P21707	Syt1	9.3	3.2	Up
O35314	Chgb	8.7	3.1	Up
Q4V7C6	Gmps	8.0	3.0	Up
Q4AE70	Carm1	7.5	2.9	Up
Q6MGD0	Cuta	7.4	2.9	Up
Q99PF5	Khsrp	7.1	2.8	Up
Q32PX7	Fubp1	7.1	2.8	Up
Q62845	Cntn4	6.6	2.7	Up
P53678	Ap3m2	6.4	2.7	Up
Q6AXT5	Rab21	6.4	2.7	Up
P02767	Ttr	5.9	2.6	Up
P0C2X9	Aldh4a1	5.7	2.5	Up
D3ZBN0	H1-5	5.6	2.5	Up
Q9Z1Y3	Cdh2	5.5	2.5	Up
P24524	Glra3	5.4	2.4	Up
P20781	Glrb	5.4	2.4	Up
P22771	Glra2	5.4	2.4	Up
P01026	C3	5.4	2.4	Up
P62747	Rhob	5.3	2.4	Up
P63319	Prkcg	5.3	2.4	Up
Q8R413	Prok2	5.2	2.4	Up
O88941	Mogs	5.1	2.4	Up
P60123	Ruvbl1	4.9	2.3	Up
Q711G3	Iah1	4.8	2.3	Up

**TABLE 2:** The detail information of the top 30 down-regulated differentially expressed proteins.

Accession	Protein	Fold change (CIBP/Sham)	Log2 fold change (CIBP/Sham)	Regulation
Q9JLA3	Uggt1	0.01	−6.2	Down
A0JPM9	Eif3j	0.02	−5.9	Down
P32362	Urod	0.04	−4.8	Down
P23514	Copb1	0.04	−4.7	Down
Q5I0D7	Pepd	0.05	−4.3	Down
P02793	Ftl1	0.05	−4.2	Down
Q7TP54	Ripor2	0.05	−4.2	Down
Q63798	Psme2	0.05	−4.2	Down
Q6IFV3	Krt15	0.07	−3.7	Down
Q63279	Krt19	0.07	−3.7	Down
Q62967	Mvd	0.08	−3.6	Down
P85125	Cavin1	0.08	−3.6	Down
Q8VHE9	Retsat	0.09	−3.6	Down
O70249	Ogg1	0.09	−3.5	Down
Q9Z173	Adgrl3	0.09	−3.5	Down
P52191	Kcnj16	0.09	−3.5	Down
P60522	Gabarapl2	0.11	−3.2	Down
P62845	Rps15	0.12	−3.1	Down
P18088	Gad1	0.12	−3.1	Down
B4F7B7	Dusp15	0.12	−3.1	Down
P63159	Hmgb1	0.12	−3.0	Down
D3ZG52	Dna2	0.12	−3.0	Down
E9PU28	Impdh2	0.13	−3.0	Down
Q6IFU8	Krt17	0.13	−3.0	Down
Q6KC51	Ablim2	0.13	−3.0	Down
P14882	Pcca	0.13	−3.0	Down
Q5M819	Psph	0.13	−3.0	Down
Q704S8	Crat	0.13	−2.9	Down
P63269	Actg2	0.13	−2.9	Down
P62738	Acta2	0.13	−2.9	Down

### Functional classification of differentially expressed protein in cancer-induced bone pain rats

**FIGURE 3:**
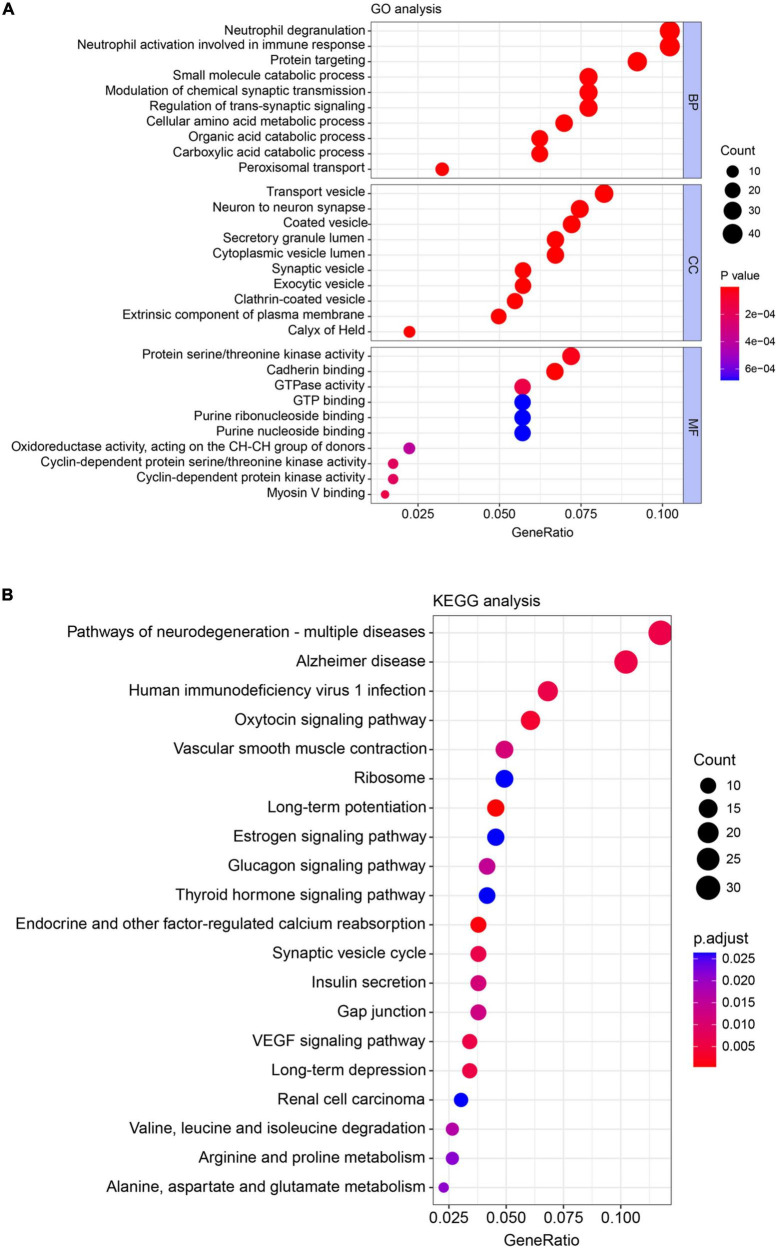
Gene ontology (GO) annotation and kyoto encyclopedia of genes and genomes (KEGG) pathway enrichment analysis of differentially expressed proteins extracted from the spinal cord. **(A)** GO analysis of the biological process (BP), cellular component (CC), and molecular function (MF) for differentially expressed proteins (DEPs). **(B)** KEGG analysis of DEPs. Count: number of DEPs related to the enriched GO or KEGG terms. Different colors indicated the range of *p-*value.

Gene ontology annotation and KEGG pathway enrichment analysis were used to deduce the function of the identified DEPs. GO terms were divided into three categories: biological process (BP), cellular component (CC), and molecular function (MF). BP category for DEPs was mainly enriched in neutrophil degranulation (GO:0043312, 41 proteins), neutrophil activation involved in immune response (GO:0002283, 41 proteins), protein targeting (GO:0006605, 35 proteins), small molecule catabolic process (GO:0044282, 30 proteins), modulation of chemical synaptic transmission (GO:0050804, 30 proteins), and regulation of *trans*-synaptic signaling (GO:0099177, 30 proteins). For the CC category, DEPs were mainly enriched in transport vesicle (GO:0030133, 34 proteins), neuron-to-neuron synapse (GO:0098984, 26 proteins), and coated vesicle (GO:0030135, 29 proteins). The top three MF categories include protein serine/threonine kinase activity (GO:0004674, 28 proteins), cadherin binding (GO:0045296, 27 proteins), and GTPase activity (GO:0003924, 23 proteins) (Figure 3A). KEGG-based functional enrichment analysis was performed to investigate the important pathways that the differentially expressed proteins were mainly related to. As shown in Figure 3B, high counts were found in the 20 identified pathways. The top three categories were pathways of neurodegeneration-multiple disease (hsa05022, 31 proteins), Alzheimer’s disease (hsa05010, 27 proteins), and human immunodeficiency virus 1 infection (hsa05170, 18 proteins).

### Proteomic changes of synapse and mitochondria in the spinal cord of cancer-induced bone pain rats

**FIGURE 4:**
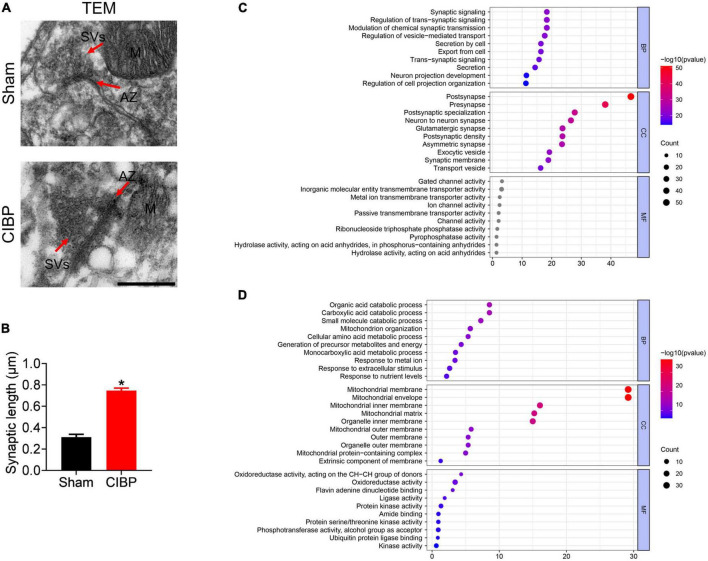
Changes in synaptic proteins and morphology. **(A)** Morphological changes in the spinal cord using transmission electron microscopy (TEM). AZ: active zone; SVs: synapse vehicles; M: mitochondria. Scale bar: 500 nm. **(B)** Relative synaptic length of sham and CIBP group. Data were expressed as the mean ± SD (*n* = 3 mice/group). *p < 0.05 vs. sham group. **(C,D)** Synaptic **(C)** and mitochondrial **(D)** proteins were classified by GO terms based on BP, CC, and MF. The color of the bar denoted –log10 (*p*-value).

Synaptic plasticity in the spinal dorsal horn is involved in pain processing (Luo et al., 2014). TEM assay was performed to confirm the morphology changes in the spinal synapse. As shown in Figure 4A, CIBP rats had more synaptic vesicles (SVs) at the active zone (AZ) and longer AZ than the sham group. The relative synaptic length of the CIBP group was increased to 1.43 ± 0.09 (*p* < 0.05 vs. sham group, Figure 4B, n = 3). Meanwhile, the mitochondrial structure was disrupted presenting as a discontinuous outer membrane and deficient cristae in the spinal cord of the CIBP group. In the sham group, the mitochondrial structure showed a whole and compact membrane (Figure 4A). Moreover, analysis of synaptic and mitochondrial proteins with DEPs showed that there were 47 up-regulated and 43 down-regulated proteins in synapse and 28 up-regulated and 38 down-regulated proteins in mitochondria. Detailed information on synaptic and mitochondrial proteins is shown in Tables 3, 4. These synaptic and mitochondrial proteins were analyzed by GO annotation. For synaptic proteins (Figure 4C), BP categories for synaptic were mainly enriched in synaptic signaling (GO: 0099536), regulation of *trans*-synaptic signaling (GO:0099177), modulation of chemical synaptic transmission (GO:0050804), and regulation of vesicle-mediated transport (GO:0060627). The CC categories for synaptic were mainly enriched in post-synapse (GO: 00098794), pre-synapse (GO: 0098793), post-synaptic specialization (GO:0099572), neuron-to-neuron synapse (GO:0098984), and glutamatergic synapse (GO:0098978). The top three MF categories for synaptic DEPs were gated channel activity (GO: 0022836), inorganic cation *trans*-membrane transporter activity (GO:0022890), and metal ion *trans*-membrane transporter activity (GO: 0046873). For mitochondrial proteins (Figure 4D), the top three BP categories were organic acid catabolic process (GO: 0016054), carboxylic acid catabolic process (GO: 0046395), and small molecule catabolic process (GO: 0044282). The top three CC categories for mitochondrial DEPs were mitochondrial membrane (GO: 0031966), mitochondrial envelope (GO: 0005740), and mitochondrial inner membrane (GO: 0005743). The MF categories for mitochondrial DEPs were mainly enriched in oxidoreductase activity, acting on the CH-CH group of donors (GO: 0016627), oxidoreductase activity (GO: 0016491), and flavin adenine dinucleotide binding (GO: 0050660).

**TABLE 3:** The information of up and down-regulated differentially expressed synaptic proteins.

Protein	Regulation	Protein	Regulation
Gsk3a	Up	Mapt	Down
Cttn	Up	Ywhag	Down
Lyn	Up	Lgals3	Down
Ywhaz	Up	Snap91	Down
Synj2bp	Up	Calb1	Down
Ywhah	Up	Septin8	Down
Arhgap44	Up	Kpna1	Down
Dcc	Up	Ntrk2	Down
Ap3m2	Up	Rab3c	Down
Snap25	Up	Atp1b2	Down
Plcb4	Up	Gad1	Down
Tprg1l	Up	Add2	Down
Cplx1	Up	Cntnap1	Down
Srcin1	Up	Rab3b	Down
Psmc5	Up	Picalm	Down
Ctnnd2	Up	Kcna3	Down
Hras	Up	Coro1a	Down
Glrb	Up	App	Down
Rab8b	Up	Add3	Down
Sncg	Up	Hpca	Down
Clta	Up	Ppp3ca	Down
Kcnc1	Up	Rab3d	Down
Cltb	Up	Mpdz	Down
Cdh2	Up	Crkl	Down
Syngap1	Up	Kras	Down
Rab3a	Up	Abi1	Down
Glra1	Up	Kcnab2	Down
Cacnb4	Up	Bcas1	Down
C3	Up	Dnajc5	Down
Cplx2	Up	Map4	Down
Lck	Up	Add1	Down
Glra2	Up	Stx6	Down
Rab8a	Up	Adgrl3	Down
Fyn	Up	Myh9	Down
Ncstn	Up	Ppp3cb	Down
Slc6a11	Up	Cdk16	Down
Trim46	Up	Slc6a9	Down
Syt5	Up	Strn	Down
Gad2	Up	Dmd	Down
Prkcb	Up	Farp1	Down
Nlgn3	Up	Wdr7	Down
Rpl10a	Up	Ap2b1	Down
Syt1	Up	Cacna2d2	Down
Cntn4	Up	Glra3	Up
Prkcg	Up	Hspb1	Up

**TABLE 4:** The information of up and down-regulated differentially expressed mitochondrial proteins.

Protein	Regulation	Protein	Regulation
Gsk3a	Up	Mapt	Down
Cttn	Up	Ywhag	Down
Lyn	Up	Lgals3	Down
Ywhaz	Up	Dna2	Down
Synj2bp	Up	Prkaa1	Down
Ywhah	Up	Etfdh	Down
Coq6	Up	Dhtkd1	Down
Ssbp1	Up	Atp5pb	Down
Tomm34	Up	Hmgcl	Down
Fgr	Up	Ndufa11	Down
Uqcrh	Up	Slc25a11	Down
Cdk1	Up	Acadvl	Down
Acot2	Up	Ppif	Down
Mfn2	Up	Prkaa2	Down
Acadl	Up	Cpox	Down
Abhd10	Up	Ppp2r2b	Down
Aldh1b1	Up	Pcca	Down
Ppp3r1	Up	Vdac3	Down
Atp5pd	Up	Crat	Down
Ivd	Up	Mecr	Down
Tamm41	Up	Pgrmc1	Down
Ass1	Up	Pdk2	Down
Ubb	Up	Slc25a3	Down
Dmac2l	Up	Fam162a	Down
Bckdhb	Up	Gatm	Down
Aldh4a1	Up	Cat	Down
Ubc	Up	Gclm	Down
Sdhaf2	Up	Tars2	Down
Cs	Down	Mrpl46	Down
Abcb7	Down	Coq9	Down
Raf1	Down	Bckdk	Down
Clu	Down	Mrpl17	Down
Dao	Down	Oat	Down

### Protein interaction networks

**FIGURE 5:**
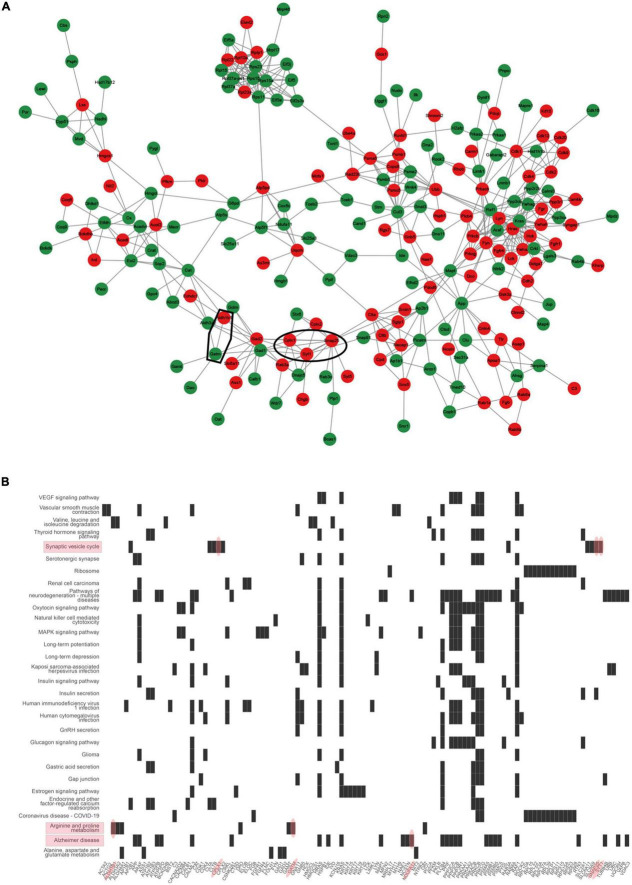
Interaction networks analyzed for differentially expressed proteins (DEPs). **(A)** STRING protein–protein interaction network of DEPs colored nodes indicates the individual protein identified. Lines between nodes represent the direct and indirect association of proteins. Red color presented up-regulated proteins. Green color presented down-regulated proteins. Circle 1: synaptic interaction proteins such as CPLX1, SNAP25, and SYT1 proteins. Circle 2: mitochondrial interaction proteins such as ALDH1B and GATM. **(B)** Heatplot presents differentially expressed proteins related to the pathway. Red ovals indicated proteins such as CPLX1, SNAP25, and SYT1 involved in the synaptic vesicle cycle pathway, and ALDH1B and GATM involved in arginine and proline metabolism.

To further investigate the protein interaction, the DEPs were further analyzed using STRING to generate an interaction network The constructed protein–protein interaction network in Figure 5A showed that chromogranin B (CHGB), complexin 1 (CPLX1), CPLX2, recombinant human DnaJ homolog subfamily C member 5 (DNAJC5), glutamic acid decarboxylase 1 (GAD1), GAD2, Ras-related protein Rab-3A (RAB3A), synaptotagmin 25 (SNAP25), syntaxin-6 (STX6), and synaptotagmin 1 (SYT1) form a protein–protein interaction network which is related to the synaptic functions. The proteins composed of the protein–protein interaction network involved in mitochondrial function were glutamate–cysteine ligase modifier subunit (GCLM), aldehyde dehydrogenase 1 family member B1 (ALDH1B), ALDH3A2, glycine amidinotransferase (GATM), guanidinoacetate N-methyltransferase (GAMT), and diamine oxidase (DAO). Meanwhile, heatplot listed proteins related to pathways (Figure 5B). In the pathway of the synaptic vesicle cycle, there were 10 proteins, including adaptor-related protein complex 2 subunit beta 1 (AP2B1), clathrin (CLTA), clathrin light chain B (CLTB), CPLX1, CPLX2, RAB3A, solute carrier family 6, member 11 (SLC6A11), SLC6A9, SNAP25, and SYT1. According to the information, ALDH1B1 and GATM belonged to the arginine and proline pathways and interacted with each other.

### Expression validation of synaptic proteins

**FIGURE 6:**
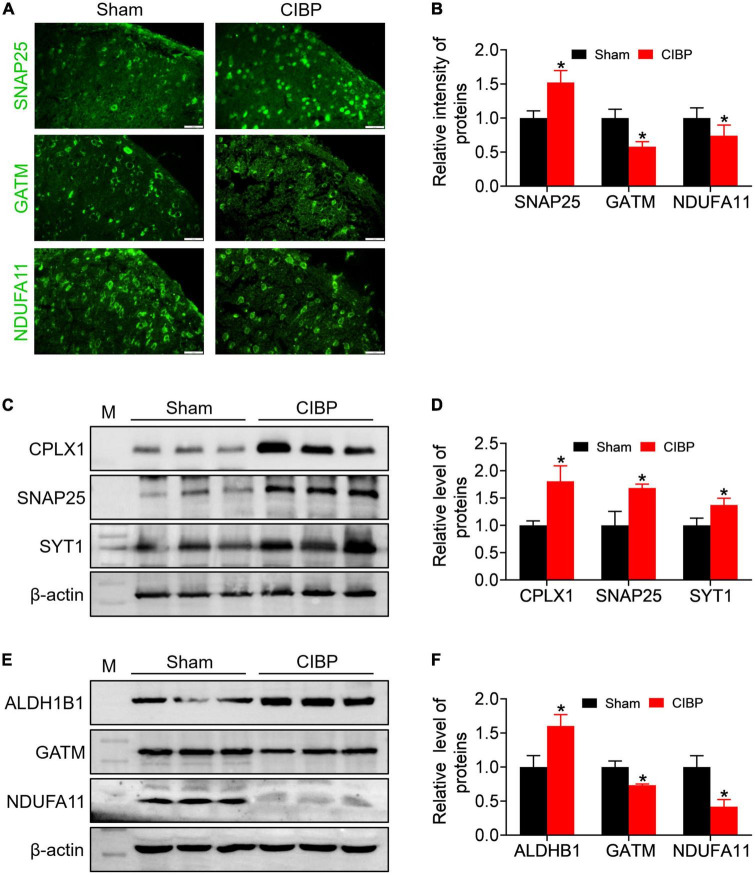
Validation of synaptic- and mitochondrial-related proteins using immunofluorescence and Western blot assay. **(A,B)** Representative immunofluorescence staining images **(A)** and quantitative analysis **(B)** for SNAP25, GATM, and NDUFA11 in the spinal cord. Scale bar = 20 μm. Data were expressed as the mean ± SD (*n* = 3 mice/group). *p < 0.05 vs. sham group. **(C,D)** Representative Western blot bands **(C)** and quantitative analysis **(D)** of CPLX1, SNAP25, and SYT1 protein in the spinal cord. Data were presented as mean ± SD (*n* = 3 mice/group). *p < 0.05 vs. sham group. **(E,F)** Representative western blot bands **(E)** and quantitative analysis **(F)** of ALDH1B, GATM, and NDUFA11 protein in spinal cord. Data were presented as mean ± SD (*n* = 3 mice/group). *p < 0.05 vs. sham group.

According to the PPI network and heatplot list, immunofluorescence and Western blot assay were used to verify the synaptic and mitochondrial proteins. In contrast to sham rats, the fluorescence intensity of SNAP25 was enhanced in the spinal dorsal horn of CIBP rats, and the relative intensity was 1.52 ± 0.17 (*p* < 0.05 vs. sham. Figures 6A,B, *n* = 3). Meanwhile, the Western blot data revealed that the expression levels of synaptic proteins CPLX1, SNAP25, and SYT1 were up-regulated in the spinal cord of CIBP rats with relative gray values as 1.80 ± 0.28, 1.68 ± 0.06, and 1.37 ± 0.12 (*p* < 0.05 vs. sham. Figures 6C,D, *n* = 3), respectively. The immunofluorescence analysis showed decreased fluorescence intensity of mitochondrial proteins, GATM, and NDUFA11 compared with the sham group (*p* < 0.05 vs. sham. Figures 6A,B, *n* = 3). In contrast to sham rats, the immunoblot band showed a changed expression of ALDH1B1, GATM, and NDUFA11 in the spinal cord of CIBP rats (*p* < 0.05 vs. sham, Figure 6E, n = 3). Relative mitochondrial protein levels of ALDH1BA, GATM, and NDUFA11 were 1.60 ± 0.16, 0.73 ± 0.01, and 1.0.41 ± 0.10, respectively (*p* < 0.05 vs. sham. Figure 6F).

## Discussion

Breast cancer is the most common cancer worldwide and is commonly diagnosed in women, the cases are expected to reach 4.4 million in 2070 (Lei S. et al., 2021). Nearly 70% of patients with metastatic breast cancer develop bone metastases. Bone metastasis causes osteolysis, spinal cord compression, and different degrees of pain (Othman et al., 2021). In this study, a rat model of CIBP was established to investigate the mechanisms of cancer-induced bone pain. We found that the CIBP rats had tibia bone structure damage, increased pain sensitivities, and impaired movement ability. These symptoms are consistent with the patients of breast cancer bone metastasis which indicated the successful construction of the CIBP rat model and was suitable for the research.

During the pathological process of CIBP, peripheral noxious signals are triggered by the metastasis of cancer cells. These noxious signals are transmitted to the spinal dorsal horn by Aδ-fibers and C-fibers and consequently induce the enhancement of synaptic plasticity and neuronal excitability (Wang et al., 2018). This disease-induced change in synaptic plasticity plays a key role in synaptic function and structure and works as a prime mechanism for pathological pain (Nesterov et al., 2021). Our data showed that the length and density of the active zone were increased in the spinal cord of CIBP rats. Proteomic analysis showed that there are 90 differentially expressed synaptic proteins in the spinal cord between CIBP rats and sham rats. Among these proteins, we verified three synaptic proteins, such as SYT1, CPLX1, and SNAP25, and confirmed increased expression of these proteins in the spinal cord of CIBP rats. SYT1 is a Ca^2+^ binding protein (Courtney et al., 2019) and functions in synaptic vesicle exocytosis *via* driving synaptic vesicle-membrane attachment (Chang et al., 2018). CPLX1 is a key synaptic protein that controls spontaneous and stimulus-evoked synaptic vesicle fusion and enhances AP-evoked synchronous release (Maximov et al., 2009). Synaptic strength is strongly reduced in the absence of CPLX1 (Chang et al., 2015). SNAP25 is a plasma membrane protein and is required for the fusion of synaptic vesicles with the plasma membrane (Huang et al., 2021). When Ca^2+^ is released into the cytoplasm, SYT1 binds with Ca^2+^ and triggers synaptic vesicle docking at the active zone. Subsequently, CPLX1 and SNAP25 promote the fusion of vesicle and pre-synaptic membranes and neurotransmitter releases into the synaptic cleft (Malsam et al., 2020). On the post-synaptic membrane, glutamate interacts and activates N-methyl-D-aspartate receptors (NMDARs) and allows the entry of Ca^2+^ into the post-synaptic neuron, which activates numerous intracellular pathways (Dhara et al., 2014). Synaptic-related proteins can be potential targets for pain therapy. Syntenin and CaM kinase 1 were identified by proteomic analysis that involved in radiotherapy of cancer pain (Park et al., 2005). Synaptic proteins such as gelsolin, apolipoprotein C1, apolipoprotein E, contactin-1, and neural cell adhesion molecule L1-like protein were altered in cerebrospinal fluid of neuropathic pain patients and contributed to pain treatment (Lind et al., 2016). Accordingly, the proteomics analysis of the spinal cord in CIBP rats provides potential targets for pain management.

In the current study, PET data revealed that the spinal cord of CIBP rats was in a hypermetabolic state, which meant high energy demands. The energy consumption depended on mitochondrial function. Mitochondria are involved in pre-synaptic axon transport, synaptic vesicle cycle, and synaptic transmission by generating ATP and regulating Ca^2+^ buffering (Zehnder et al., 2021). High energy demands drive mitochondrial fission and cause mitochondrial fragment and dysfunction (Gokhale et al., 2021). In our study, we found that the mitochondrial structure was damaged, and 66 mitochondrial proteins overlapped with differential expressed proteins. Among them, the expressions of NDUFA11, ALDH1B1, and GATM were confirmed to increase in the spinal cord of CIBP rats in our validation experiments. NDUFA11 is a subunit of complex I for the mitochondrial respiratory chain (Grivennikova et al., 2020), which functions on the oxidization of NADH to NAD^+^ and then transfers electrons to the carrier ubiquinone (Kushnareva et al., 2002). NDUFA11 is required for balancing the respiratory chain, mitochondrial morphology, and development (Knapp-Wilson et al., 2021). NDUFA11 knockdown stimulates the dissociation of respirasome and reduces the activity of complexes I, III, and IV (Jang and Javadov, 2018). ALDHs belong to a superfamily of NAD(P)^+^-dependent enzymes that catalyze the oxidation of endogenous and exogenous aldehydes to their corresponding acids. ALDH1B1 is a mitochondrial ALDH that metabolizes a wide range of aldehyde substrates, including acetaldehyde and products of lipid peroxidation (Chen et al., 2011). ALDH1B1 is overexpressed in various cancers (Zhu et al., 2022) and is tightly associated with tumorigenesis and therapy resistance (Feng et al., 2022). Mitochondrial enzyme GATM has a role in creatine biosynthesis, acting as a dynamic reservoir of high-energy phosphate and playing an essential role in the energy metabolism of nerve tissues (Courtoy and Henriet, 2018). GATM mutation impairs mitochondrial turnover and leads to increased production of reactive oxygen species (Carney, 2018). These data suggested that mitochondrial dysfunction probably is a key factor for cancer pain.

Researches indicate that damaged mitochondria increase the generation of reactive oxygen species and mediated the activation of inflammatory response (Ke et al., 2017; Park et al., 2017). Neuroinflammation in the spinal cord is a cardinal feature of pain, characterized by leukocyte infiltration, glial activation, and the release of proinflammatory cytokines and chemokines (Ji et al., 2016). Our data showed that spinal proteins related to neutrophil-mediated immunity were differentially expressed in sham and CIBP rats, and this was validated by H&E staining, which showed that leukocyte infiltration increased in the spinal cord of CIBP rats. Taken together, these data suggested that targeting synaptic- and mitochondrial-related proteins is a strategy for the therapy of neuroinflammation-related pain.

## Conclusion

**FIGURE 7:**
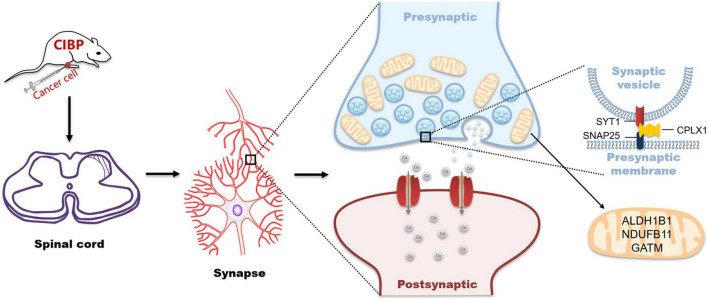
Schematic representation for changes in synaptic- and mitochondrial-related proteins in the spinal cord during cancer pain processing.

In summary, our findings revealed that synaptic- and mitochondrial-related proteins in the spinal cord potentially involve in cancer-induced bone pain (Figure 7). Our data provide evidence for understanding the mechanism of cancer-induced bone pain and the theoretical basis for new therapeutic approaches.

## Data availability statement

The original contributions presented in this study are publicly available. This data can be found here: https://www.iprox.cn/, IPX0004820000.

## Ethics statement

This animal study was reviewed and approved by Ethics Committee of Hubei University of Science and Technology.

## Author contributions

HY, JW, SZ, YH, DL, MX, and HZ performed experiments, collected and analyzed the data, and drafted the manuscript. MX and HZ designed the experiments, analyzed the data, and wrote the manuscript. All authors read and approved the final manuscript.

## Footnotes

1
https://string-db.org/

## References

[B1] Ban J. Fock V. Aryee D. Kovar H. (2021). Mechanisms, diagnosis and treatment of bone metastases. *Cells* 10:2944. 10.3390/cells10112944 34831167 PMC8616226

[B2] Bosma R. L. Mojarad E. A. Leung L. Pukall C. Staud R. Stroman P. W. (2016). FMRI of spinal and supra-spinal correlates of temporal pain summation in fibromyalgia patients. *Hum. Brain Mapp.* 37 1349–1360. 10.1002/hbm.23106 26749315 PMC4783193

[B3] Carney E. F. (2018). GATM mutations cause mitochondrial abnormalities and kidney failure. *Nat. Rev. Nephrol.* 14:414. 10.1038/s41581-018-0017-3 29695752

[B4] Chang S. Reim K. Pedersen M. Neher E. Brose N. Taschenberger H. (2015). Complexin stabilizes newly primed synaptic vesicles and prevents their premature fusion at the mouse calyx of held synapse. *J. Neurosci.* 35 8272–8290. 10.1523/JNEUROSCI.4841-14.2015 26019341 PMC6605347

[B5] Chang S. Trimbuch T. Rosenmund C. (2018). Synaptotagmin-1 drives synchronous Ca2+-triggered fusion by C2B-domain-mediated synaptic-vesicle-membrane attachment. *Nat. Neurosci.* 21 33–40. 10.1038/s41593-017-0037-5 29230057 PMC5742540

[B6] Chen H. S. Holmes N. Liu J. Tetzlaff W. Kozlowski P. (2017). Validating myelin water imaging with transmission electron microscopy in a rat spinal cord injury model. *Neuroimage* 153 122–130. 10.1016/j.neuroimage.2017.03.065 28377211

[B7] Chen Y. Orlicky D. J. Matsumoto A. Singh S. Thompson D. C. Vasiliou V. (2011). Aldehyde dehydrogenase 1B1 (ALDH1B1) is a potential biomarker for human colon cancer. *Biochem. Biophys. Res. Commun.* 405 173–179. 10.1016/j.bbrc.2011.01.002 21216231 PMC3112362

[B8] Courtney N. A. Bao H. Briguglio J. S. Chapman E. R. (2019). Synaptotagmin 1 clamps synaptic vesicle fusion in mammalian neurons independent of complexin. *Nat. Commun.* 10:4076. 10.1038/s41467-019-12015-w 31501440 PMC6733930

[B9] Courtoy P. J. Henriet P. (2018). GATM mutations cause a dominant fibrillar conformational disease in mitochondria-when eternity kills. *J. Am. Soc. Nephrol.* 29 1787–1789. 10.1681/ASN.2018040450 29789432 PMC6050931

[B10] Dhara M. Yarzagaray A. Schwarz Y. Dutta S. Grabner C. Moghadam P. K. (2014). Complexin synchronizes primed vesicle exocytosis and regulates fusion pore dynamics. *J. Cell Biol.* 204 1123–1140. 10.1083/jcb.201311085 24687280 PMC3971750

[B11] Dixon W. J. (1980). Efficient analysis of experimental observations. *Annu. Rev. Pharmacol. Toxicol.* 20 441–462. 10.1146/annurev.pa.20.040180.002301 7387124

[B12] Doncheva N. T. Morris J. H. Gorodkin J. Jensen L. J. (2019). Cytoscape stringapp: Network analysis and visualization of proteomics data. *J. Proteome Res.* 18 623–632. 10.1021/acs.jproteome.8b00702 30450911 PMC6800166

[B13] Dong Z. B. Wang Y. J. Wan W. J. Wu J. Wang B. J. Zhu H. L. (2022). Resveratrol ameliorates oxaliplatin-induced neuropathic pain via anti-inflammatory effects in rats. *Exp. Ther. Med.* 24:586. 10.3892/etm.2022.11523 35949346 PMC9353538

[B14] Ellingson H. M. Vanderah T. W. (2020). Potential therapeutic treatments of cancer-induced bone pain. *Curr. Opin. Support. Palliat. Care* 14 107–111. 10.1097/SPC.0000000000000496 32349095 PMC7815248

[B15] Feng Z. Hom M. E. Bearrood T. E. Rosenthal Z. C. Fernández D. Ondrus A. E. (2022). Targeting colorectal cancer with small-molecule inhibitors of ALDH1B1. *Nat. Chem. Biol.* 18 1065–1075. 10.1038/s41589-022-01048-w 35788181 PMC9529790

[B16] Garcia K. Wray J. K. Kumar S. (2022). *Spinal cord stimulation.* Treasure Island, FL: In StatPearls. 31985947

[B17] Glithero C. (2020). The challenges of managing bone pain in cancer. *Ulster Med. J.* 89 7–10. 32218619 PMC7027188

[B18] Gokhale A. Lee C. E. Zlatic S. A. Freeman A. Shearing N. Hartwig C. (2021). Mitochondrial proteostasis requires genes encoded in a neurodevelopmental syndrome locus. *J. Neurosci.* 41 6596–6616. 10.1523/JNEUROSCI.2197-20.2021 34261699 PMC8336702

[B19] Grivennikova V. G. Gladyshev G. V. Vinogradov A. D. (2020). Deactivation of mitochondrial NADH:ubiquinone oxidoreductase (respiratory complex I): Extrinsically affecting factors. *Biochim. Biophys. Acta Bioenerg.* 1861:148207. 10.1016/j.bbabio.2020.148207 32315625

[B20] Huang Q. Lian C. Dong Y. Zeng H. Liu B. Xu N. (2021). SNAP25 inhibits glioma progression by regulating synapse plasticity via GLS-mediated glutaminolysis. *Front. Oncol.* 11:698835. 10.3389/fonc.2021.698835 34490096 PMC8416623

[B21] Jang S. Javadov S. (2018). Elucidating the contribution of ETC complexes I and II to the respirasome formation in cardiac mitochondria. *Sci. Rep.* 8:17732. 10.1038/s41598-018-36040-9 30531981 PMC6286307

[B22] Ji R. R. Chamessian A. Zhang Y. Q. (2016). Pain regulation by non-neuronal cells and inflammation. *Science* 354 572–577. 10.1126/science.aaf8924 27811267 PMC5488328

[B23] Julius D. Basbaum A. I. (2001). Molecular mechanisms of nociception. *Nature* 413 203–210. 10.1038/35093019 11557989

[B24] Kapoor R. Saxena A. K. Vasudev P. Sundriyal D. Kumar A. (2021). Cancer induced bone pain: Current management and future perspectives. *Med. Oncol.* 38:134. 10.1007/s12032-021-01587-7 34581894

[B25] Kawada K. Iwamoto M. Sakai Y. (2016). Mechanisms underlying 18F-fluorodeoxyglucose accumulation in colorectal cancer. *World J. Radiol.* 8 880–886. 10.4329/wjr.v8.i11.880 27928469 PMC5120247

[B26] Ke C. Gao F. Tian X. Li C. Shi D. He W. (2017). Slit2/robo1 mediation of synaptic plasticity contributes to bone cancer pain. *Mol. Neurobiol.* 54 295–307. 10.1007/s12035-015-9564-9 26738857

[B27] Knapp-Wilson A. Pereira G. C. Buzzard E. Ford H. C. Richardson A. Corey R. A. (2021). Maintenance of complex I and its supercomplexes by NDUF-11 is essential for mitochondrial structure, function and health. *J. Cell Sci.* 134:jcs258399. 10.1242/jcs.258399 34106255 PMC8277142

[B28] Kornelsen J. Mackey S. (2007). Potential clinical applications for spinal functional MRI. *Curr. Pain Headache Rep.* 11 165–170. 10.1007/s11916-007-0186-4 17504642 PMC2914611

[B29] Kuner R. (2010). Central mechanisms of pathological pain. *Nat. Med.* 16 1258–1266. 10.1038/nm.2231 20948531

[B30] Kushnareva Y. Murphy A. N. Andreyev A. (2002). Complex I-mediated reactive oxygen species generation: Modulation by cytochrome c and NAD(P)+ oxidation-reduction state. *Biochem. J.* 368(Pt 2) 545–553. 10.1042/BJ20021121 12180906 PMC1222999

[B31] Laakso H. Lehto L. J. Paasonen J. Salo R. Canna A. Lavrov I. (2021). Spinal cord fMRI with MB-SWIFT for assessing epidural spinal cord stimulation in rats. *Magn. Reson. Med.* 86 2137–2145. 10.1002/mrm.28844 34002880 PMC8360072

[B32] Lei S. Zheng R. Zhang S. Wang S. Chen R. Sun K. (2021a). Global patterns of breast cancer incidence and mortality: A population-based cancer registry data analysis from 2000 to 2020. *Cancer Commun. (Lond)* 41 1183–1194. 10.1002/cac2.12207 34399040 PMC8626596

[B33] Lei T. Qian H. Lei P. Hu Y. (2021b). Ferroptosis-related gene signature associates with immunity and predicts prognosis accurately in patients with osteosarcoma. *Cancer Sci.* 112 4785–4798. 10.1111/cas.15131 34506683 PMC8586685

[B34] Li H. Xu H. Wen W. Wu L. Xu M. Luo J. (2020). Thiamine deficiency causes long-lasting neurobehavioral deficits in mice. *Brain Sci.* 10:565. 10.3390/brainsci10080565 32824629 PMC7464042

[B35] Li M. Y. Ding J. Q. Tang Q. Hao M. M. Wang B. H. Wu J. (2019). SIRT1 activation by SRT1720 attenuates bone cancer pain via preventing Drp1-mediated mitochondrial fission. *Biochim. Biophys. Acta Mol. Basis Dis.* 1865 587–598. 10.1016/j.bbadis.2018.12.017 30579931

[B36] Lind A. L. Emami Khoonsari P. Sjödin M. Katila L. Wetterhall M. Gordh T. (2016). Spinal cord stimulation alters protein levels in the cerebrospinal fluid of neuropathic pain patients: A proteomic mass spectrometric analysis. *Neuromodulation* 19 549–562. 10.1111/ner.12473 27513633

[B37] Liu M. Cheng X. Yan H. Chen J. Liu C. Chen Z. (2021). MiR-135-5p alleviates bone cancer pain by regulating astrocyte-mediated neuroinflammation in spinal cord through JAK2/STAT3 signaling pathway. *Mol. Neurobiol.* 58 4802–4815. 10.1007/s12035-021-02458-y 34176097

[B38] Luo C. Kuner T. Kuner R. (2014). Synaptic plasticity in pathological pain. *Trends Neurosci.* 37 343–355. 10.1016/j.tins.2014.04.002 24833289

[B39] Malsam J. Bärfuss S. Trimbuch T. Zarebidaki F. Sonnen A. F. Wild K. (2020). Complexin suppresses spontaneous exocytosis by capturing the membrane-proximal regions of VAMP2 and SNAP25. *Cell Rep.* 32:107926. 10.1016/j.celrep.2020.107926 32698012 PMC7116205

[B40] Mathavan N. Koopman J. Raina D. B. Turkiewicz A. Tägil M. Isaksson H. (2019). 18F-fluoride as a prognostic indicator of bone regeneration. *Acta Biomater.* 90 403–411. 10.1016/j.actbio.2019.04.008 30965143

[B41] Maximov A. Tang J. Yang X. Pang Z. P. Südhof T. C. (2009). Complexin controls the force transfer from SNARE complexes to membranes in fusion. *Science* 323 516–521. 10.1126/science.1166505 19164751 PMC3235366

[B42] Nesterov S. Chesnokov Y. Kamyshinsky R. Panteleeva A. Lyamzaev K. Vasilov R. (2021). Ordered clusters of the complete oxidative phosphorylation system in cardiac mitochondria. *Int. J. Mol. Sci.* 22:1462. 10.3390/ijms22031462 33540542 PMC7867189

[B43] Othman A. Winogradzki M. Lee L. Tandon M. Blank A. Pratap J. (2021). Bone metastatic breast cancer: Advances in cell signaling and autophagy related mechanisms. *Cancers* 13:4310. 10.3390/cancers13174310 34503118 PMC8431094

[B44] Paolini F. Ferini G. Bonosi L. Costanzo R. Brunasso L. Benigno U. E. (2022). Spinal cord stimulation to treat unresponsive cancer pain: A possible solution in palliative oncological therapy. *Life (Basel)* 12:554. 10.3390/life12040554 35455045 PMC9025741

[B45] Park E. S. Ahn J. M. Jeon S. M. Cho H. J. Chung K. M. Cho J. Y. (2017). Proteomic analysis of the dorsal spinal cord in the mouse model of spared nerve injury-induced neuropathic pain. *J. Biomed. Res.* 31 494–502. 10.7555/JBR.31.20160122 28866658 PMC6307668

[B46] Park H. C. Seong J. An J. H. Kim J. Kim U. J. Lee B. W. (2005). Alteration of cancer pain-related signals by radiation: Proteomic analysis in an animal model with cancer bone invasion. *Int. J. Radiat. Oncol. Biol. Phys.* 61 1523–1534. 10.1016/j.ijrobp.2004.12.070 15817359

[B47] Shi X. Bai H. Wang J. Wang J. Huang L. He M. (2021). Behavioral assessment of sensory, motor, emotion, and cognition in rodent models of intracerebral hemorrhage. *Front. Neurol.* 12:667511. 10.3389/fneur.2021.667511 34220676 PMC8248664

[B48] Siegel R. L. Miller K. D. Fuchs H. E. Jemal A. (2022). Cancer statistics, 2022. *CA Cancer J. Clin.* 72 7–33. 10.3322/caac.21708 35020204

[B49] Tilley D. M. Lietz C. B. Cedeno D. L. Kelley C. A. Li L. Vallejo R. (2021). Proteomic modulation in the dorsal spinal cord following spinal cord stimulation therapy in an in vivo neuropathic pain model. *Neuromodulation* 24 22–32. 10.1111/ner.13103 32157770 PMC7484326

[B50] von Leden R. E. Selwyn R. G. Jaiswal S. Wilson C. M. Khayrullina G. Byrnes K. R. (2016). (18)F-FDG-PET imaging of rat spinal cord demonstrates altered glucose uptake acutely after contusion injury. *Neurosci. Lett.* 621 126–132. 10.1016/j.neulet.2016.04.027 27084688 PMC5018212

[B51] Wang K. Donnelly C. R. Jiang C. Liao Y. Luo X. Tao X. (2021). STING suppresses bone cancer pain via immune and neuronal modulation. *Nat. Commun.* 12:4558. 10.1038/s41467-021-24867-2 34315904 PMC8316360

[B52] Wang K. Gu Y. Liao Y. Bang S. Donnelly C. R. Chen O. (2020). PD-1 blockade inhibits osteoclast formation and murine bone cancer pain. *J. Clin. Invest.* 130 3603–3620. 10.1172/JCI133334 32484460 PMC7324182

[B53] Wang L. Chen S. R. Ma H. Chen H. Hittelman W. N. Pan H. L. (2018). Regulating nociceptive transmission by VGluT2-expressing spinal dorsal horn neurons. *J. Neurochem.* 147 526–540. 10.1111/jnc.14588 30203849 PMC6263733

[B54] Yanagisawa Y. Furue H. Kawamata T. Uta D. Yamamoto J. Furuse S. (2010). Bone cancer induces a unique central sensitization through synaptic changes in a wide area of the spinal cord. *Mol. Pain* 6 38–51. 10.1186/1744-8069-6-38 20602757 PMC3020802

[B55] Yao F. D. Yang J. Q. Huang Y. C. Luo M. P. Yang W. J. Zhang B. (2019). Antinociceptive effects of ginsenoside Rb1 in a rat model of cancer-induced bone pain. *Exp. Ther. Med.* 17 3859–3866. 10.3892/etm.2019.7404 30988771 PMC6447891

[B56] Yu W. Qin X. Zhang Y. Qiu P. Wang L. Zha W. (2020). Curcumin suppresses doxorubicin-induced cardiomyocyte pyroptosis via a PI3K/Akt/mTOR-dependent manner. *Cardiovasc. Diagn. Ther.* 10 752–769. 10.21037/cdt-19-707 32968631 PMC7487371

[B57] Zaja̧czkowska R. Kocot-Kêpska M. Leppert W. Wordliczek J. (2019). Bone pain in cancer patients: Mechanisms and current treatment. *Int. J. Mol. Sci.* 20:6047. 10.3390/ijms20236047 31801267 PMC6928918

[B58] Zehnder T. Petrelli F. Romanos J. De Oliveira Figueiredo E. C. Lewis T. L. Jr. Déglon N. (2021). Mitochondrial biogenesis in developing astrocytes regulates astrocyte maturation and synapse formation. *Cell Rep.* 35:108952. 10.1016/j.celrep.2021.108952 33852851

[B59] Zhang X. Karim M. Hasan M. M. Hooper J. Wahab R. Roy S. (2022). Cancer-on-a-chip: Models for studying metastasis. *Cancers* 14:648. 10.3390/cancers14030648 35158914 PMC8833392

[B60] Zhao Y. Sun L. Zhu G. Dovichi N. J. (2016). Coupling capillary zone electrophoresis to a q exactive hf mass spectrometer for top-down proteomics: 580 proteoform identifications from yeast. *J. Proteome Res.* 15 3679–3685. 10.1021/acs.jproteome.6b00493 27490796 PMC5075424

[B61] Zhu T. He J. Liu Y. Deng K. Zuo J. Ai X. (2022). ALDH1B1 predicts poor survival for locally advanced nasopharyngeal carcinoma patients. *Transl. Cancer Res.* 11 382–391. 10.21037/tcr-21-1979 35281423 PMC8904955

